# Serum IL-17 and TNFα as prognostic biomarkers in systemic sclerosis patients: a prospective study

**DOI:** 10.1007/s00296-023-05499-9

**Published:** 2023-12-05

**Authors:** Joanna Kosałka-Węgiel, Sabina Lichołai, Renata Pacholczak-Madej, Sylwia Dziedzina, Mamert Milewski, Piotr Kuszmiersz, Anna Korona, Jolanta Gąsior, Aleksandra Matyja-Bednarczyk, Helena Kwiatkowska, Lech Zaręba, Andżelika Siwiec-Koźlik, Paweł Koźlik-Siwiec, Anita Wach, Weronika Pociej-Marciak, Marek Sanak, Jacek Musiał, Stanisława Bazan-Socha, Mariusz Korkosz

**Affiliations:** 1 Rheumatology and Immunology Clinical Department, University Hospital, Krakow, Poland; 2 Department of Rheumatology and Immunology, Jagiellonian University Medical College, Jakubowskiego 2 Str., 30-688 Kraków, Poland; 3 2nd Department of Internal Medicine, Jagiellonian University Medical College, Jakubowskiego 2 Str., 30-688 Kraków, Poland; 4 Department of Chemotherapy, The District Hospital, Sucha Beskidzka, Poland; 5 Department of Anatomy, Jagiellonian University Medical College, Krakow, Poland; 6 Department of Dermatology, University Hospital, Krakow, Poland; 7 Interdisciplinary Centre for Computational Modelling, College of Natural Sciences, University of Rzeszow, Rzeszow, Poland; 8 Department of Haematology, University Hospital, Krakow, Poland; 9 Division of Ophthalmology and Ocular Oncology, Department of Ophthalmology, Faculty of Medicine, Jagiellonian University Medical College, Krakow, Poland; 10 Department of Ophthalmology and Ocular Oncology, University Hospital, Krakow, Poland

**Keywords:** Systemic sclerosis, Interleukin 17, Tumor necrosis factor-alpha, Cancer, Biomarkers, Cytokines

## Abstract

Recent reports have demonstrated that endothelial injury is critical in the pathogenesis of systemic sclerosis (SSc) and is associated with increased levels of circulating inflammatory biomarkers. This study aims to analyze the serum concentrations of selected cytokines and evaluate their relationship with SSc clinics and the long-term course of the disease. This study included 43 SSc patients and 24 matched healthy controls. In both groups, we measured serum levels of inflammatory cytokines related to the inflammatory response, such as tumor necrosis factor (TNF)α, interferon (IFN)γ, interleukin (IL)-4, IL-6, IL-10, and IL-17, and fibroblast activation protein (FAP). Additionally, in SSc patients, we evaluated the presence of four single nucleotide polymorphisms (SNPs) located in the promotor region of the *TNFA* gene, namely rs361525, rs1800629, rs1799964, and rs1799724, which might be related to increased TNFα concentrations. The main aim consisted of associating inflammatory cytokines with (1) clinical disease characteristics and (2) longitudinal observation of survival and cancer prevalence. SSc patients were characterized by a 17% increase in serum TNFα. There was no other difference in serum cytokines between the studied groups and diffuse vs. limited SSc patients. As expected, evaluated serum cytokines correlated with inflammatory biomarkers (e.g., IL-6 and C-reactive protein). Interestingly, patients with higher IL-17 had decreased left ventricle ejection fraction. During the median 5-year follow-up, we recorded four cases of neoplastic diseases (lung cancer in two cases, squamous cell carcinoma of unknown origin, and breast cancer with concomitant multiple myeloma) and nine deaths. The causes of death included lung cancer (*n* = 2), renal crisis (*n* = 1), multiple-organ failure (*n* = 1), and unknown reasons in five cases. Surprisingly, higher TNFα was associated with an increased cancer prevalence, while elevated IL-17 with death risk in the follow-up. Furthermore, the AG rs361525 genotype referred to higher TNFα levels than GG carriers. Both AG rs361525 and CT rs1799964 genotypes were associated with increased cancer risk. Higher serum concentrations of TNFα characterize the SSc patients, with the highest values associated with cancer. On the other hand, increased IL-17 in peripheral blood might predict poor SSc prognosis. Further research is needed to validate these findings.

## Introduction

Systemic sclerosis (SSc) is a complex multiorgan autoimmune disease characterized by widespread peripheral microvascular injury leading to progressive skin and internal organs fibrosis [1–6]. The etiology of SSc is still unclear, but genetic and environmental factors, among others, are likely implicated [7]. For example, higher susceptibility had been reported in females and Afro-Americans, as well as in the USA and Australia, compared to Japan and Europe [7]. The significant causes of mortality in SSc are those related to lung involvement, including pulmonary hypertension (PAH) and interstitial lung disease (ILD) [4]. Other SSc manifestations, such as cardiac fibrosis and renal and gastrointestinal tract involvement, are also essentially severe but rarely lead to death [4, 5, 8, 9]. Despite improving overall survival, non-SSc-related deaths, particularly malignancies, have become more prevalent, accounting for 5 to 31% of deaths in that group of patients [4, 9–12]. Cancer incidence among SSc patients is significantly higher than in the general population and other rheumatic diseases [4, 13]. Lung, breast, esophageal, ENT, skin, and primary liver cancers and hematological disorders are reported to be the most common, although the results vary [4, 14–19]. Some data suggest that a higher cancer rate in SSc is associated with diffuse cutaneous involvement (dcSSc), persistent systemic hyperinflammatory state, immunosuppressants use, cigarette smoking, male gender, and some genetic factors, including specific tumor necrosis factor (TNF)α promoter variants [1]. Notably, ILD is a known risk factor for lung cancer, accounting for approximately one-third of the cancers seen in SSc patients [15, 20]. Furthermore, the presence of specific SSc-related antinuclear autoantibodies might also be of importance [4, 13–15, 21], however, the ties of neoplasms with antibodies to topoisomerase-I (Scl-70) and ribonucleic acid (RNA) polymerase I/III remains conflicting [14, 15, 18, 22–24].

Several cytokines, including TNFα, interleukin (IL)-6, IL-17, IL-4, interferon (IFN)γ, and IL-10, have been implicated as potential mediators of vasculopathy and fibrosis in SSc [25–31]. TNFα activates the vascular endothelium, and augments type I collagen production by fibroblasts, leading to fibrotic response; thus may be important in SSc pathogenesis [1]. Moreover, TNFα potentiates the platelet-activating factor (PAF)-induced vasoconstriction in the pulmonary circulation and plays a role in inflammation and vascular injury depending on low-density lipoprotein receptor 1 (LOX-1) activation [1]. IL-17 is a pro-inflammatory cytokine produced by a subset of T helper cells termed Th17, which appears to act not directly on immune cells but stimulates stromal cells such as endothelial and epithelial cells and fibroblasts to secrete other immunomodulatory factors [25]. Fibroblast activated by IL-17 can support the growth and differentiation of immune cells [25]. IL-17 also stimulates neutrophils to produce extracellular traps, augmenting local inflammatory response [25]. However, emerging data points to the dual IL-17 functioning by enhancing or alleviating immune and inflammatory responses, depending on disease settings [25]. Fibroblast activation protein (FAP) is a novel cell-surface serine protease acting on various extracellular matrix components [32]. FAP is highly upregulated, especially on fibroblasts, at sites of active tissue remodeling, including malignancy, but is also associated with non-malignant conditions, such as SSc or wound healing [32, 33].

Considering the higher rate of malignancy in SSc and the fact that the exact pathophysiology of carcinogenesis in SSc remains poorly understood [15, 20] we aim to investigate the impact of selected serum cytokine levels, including TNFα and FAP, on clinical SSc manifestations, as well as cancer and death prevalence during a five year of follow-up.

## Patients and methods

This study is a continuation of our previous reports (https://advances.umw.edu.pl/en/ahead-of-print/168724/ and https://www.mdpi.com/2075-1729/12/5/698); however, in this project, we analyzed new, previously not evaluated cytokines, including TNFα, IL-6, IL-17, IL-4, IFNγ, and IL-10, and assessed their associations with SSc clinics and TNFα polymorphisms.

### Patients

The patients were diagnosed with SSc according to the 2013 European League Against Rheumatism/American College of Rheumatology (EULAR/ACR) classification criteria [34]. Detailed demographical, clinical, and laboratory characteristics of enrolled systemic sclerosis patients and controls are provided in our previous publication [35] and shortly described here. Briefly, the evaluated clinical manifestations included: digital ulcers, abnormal nailfold capillaries, teleangiectasia, Raynaud’s phenomenon, dysphagia, ILD, PAH, and renal crisis [35]. Abnormal nailfold capillaries were confirmed by qualified medical staff performing image analysis of videographs of nailfold capillaries [36]. Telangiectasia was defined as macroscopically visible dilated skin vessels occurring primarily on the hands and face [37]. Raynaud’s phenomenon was defined as episodic vasospasm of fingers and toes in response to cold with tri-phasic color change [38]. Dysphagia was diagnosed on the basis of clinical symptoms and/or imaging examinations (i. e. videofluoroscopy) [39]. The presence of SSc-associated ILD was evaluated based on chest high-resolution computed tomography (HRCT). Spirometry and bronchial reversibility test (after 400 μg of salbuterol) were assessed according to the standards of the American Thoracic Society, using a Jaeger MasterLab spirometer (Jaeger-Toennies GmbH, Hochberg, Germany) [40]. A high echocardiographic probability of pulmonary hypertension was defined as a pulmonary artery systolic pressure > 45 mmHg measured in transthoracic echocardiography [1, 2]. This non-invasive method has 97% specificity as compared to the right heart catheterization, considered a gold standard for the diagnosis of PAH [41]. Scleroderma renal crisis was defined as a new renal insufficiency with or without arterial hypertension if not explained by reasons other than SSc [35]. We also analyzed other comorbidities, such as arterial hypertension, diabetes mellitus, and hypercholesterolemia. They all were defined in our previous article. The treatment modalities included steroids, azathioprine, methotrexate, mycophenolate mofetil, and cyclophosphamide, currently or in the past.

Candidates for the study were excluded if they had an active acute or chronic infection, including viral hepatitis, other chronic inflammatory diseases (e.g. rhinosinusitis), heart failure with left ventricular ejection fraction (LVEF) ≤ 40% [42], active cancer, kidney failure, defined as estimated glomerular filtration rate (eGFR, using Modification of Diet in Renal Disease formula) < 45 mL/min/1.73 m^2^, or if they were pregnant, breastfeeding or still six months after delivery. SSc cases were followed up for a median of 5 years to record cancer and death incidence for their potential associations with the cytokines levels at baseline.

The study was conducted in accordance with the Declaration of Helsinki and approved by the Bioethics Committee at the Jagiellonian University Medical College (approval code KBET/235/B/2013 and date of approval 26 September 2013). All study participants received a thorough explanation of the methodology and safety protocol before giving written consent for their inclusion.

### Laboratory analysis

Complete blood cell count, plasma fibrinogen, lipid profile, and serum levels of C-reactive protein (CRP), alanine and aspartate transaminases, bilirubin, alkaline phosphatase, urea, and creatinine with estimated glomerular filtration rate (eGFR, using Modification of Diet in Renal Disease formula), antinuclear antibodies (ANA) were measured in all patients. Laboratory analysis was defined in our previous Manuscript [35]. Genotyping of TNFA gene polymorphisms (rs361525, rs1800629, rs1799724, and rs1799964) is described in the previous Manuscript [1].

### Serum cytokines

Serum levels of FAP, IFNγ, IL-4, IL-6, IL-10, IL-17, and TNF α were measured using Procarta Luminex Immunoassays (eBioscience, San Diego, CA) according to the recommended protocol on the MAGPIX instrument (Luminex Corp., Austin, TX) and xPonent software (Luminex). Results below the detection threshold were assigned the values of lower assay limit.

### Statistical analyses

The data were analysed using STATISTICA Tibco 13.3 software and R software. According to the Shapiro–Wilk test, all continuous variables distribution departed from the normal one, so they were presented in the manuscript as median with [Q1; Q3] interval. Mann–Whitney *U* test was used to compare variables and assess the statistical significance of the rank distribution. One-way covariance analysis (ANCOVA) was performed to adjust for potential confounders, including sex, age, and body mass index (BMI). Categorical variables were reported as percentages and compared using the Chi^2^ test. The relationship between continuous variables was evaluated using the Spearman rank correlation test. Multiple linear regression models were used to determine independent determinants of serum cytokines, with *R*^2^ assessed as a measure of the variance. The backward stepwise regression method was used to select predictors for the regression model. Odds ratios (OR) with a 95% confidence interval (CI) were calculated using cut-off points evaluated based on receiver operating characteristic (ROC) curves, using the Youden method. Clusters analysis was performed using the k-means method based on laboratory test results, including cytokine profiles, to indicate disease phenotypes. Statistical significance was set at *p* < 0.05. Genotype frequency of each population was tested for Hardy–Weinberg equilibrium, and the differences between the observed and expected numbers of each genotype were compared using a goodness-of-fit chi-square test. Hardy–Weinberg equilibrium was assumed for *p* > 0.05.

## Results

### Patients’ characteristics

The demographic and clinical characteristics of the enrolled patients are summarized in Table 1. Briefly, at enrolment, the median duration of the disease was 4 (1–11) years, and most patients (*n* = 26, 60.5%) were diagnosed with the diffuse type of the disease. Raynaud’s phenomenon was the most frequent clinical sign reported in 95.3% of cases. SSc-ILD was found in nearly 70% of patients, while other manifestations, including dysphagia, probably PAH, and digital ulcers, were reported much less frequent (Table 1). All but one patient had eGFR > 60 ml/min/1.73m^2^. At enrolment, 16 SSc patients (37.2%) were treated with steroids, and 7 individuals (16.3%) currently received immunosuppressive drugs, such as cyclophosphamide (3 patients), methotrexate (2 patients), mycophenolate mofetil (1 patient), and azathioprine (1 patient). The median daily methylprednisolone dose was 0 (0–4) mg (Table 1). Other medications used, such as calcium channel blockers, angiotensin-converting enzyme inhibitors or angiotensin receptor antagonists, and statins, are also listed in Table 1.

**Table 1:** Demographic, clinical, and immunological characteristics of systemic sclerosis patients, *n* = 43

Age, years	59 (44–65)
Male gender, *n*(%)	10 (23.3)
Body mass index, kg/m^2^	23.6 (22.2–26.1)
Smoking, currently or in the past, *n*(%)	15 (34.9)
Smoking, packs/years	0 (0–5)
Duration of the disease, years	6 (1–11)
Limited disease, *n*(%)	17 (39.5)
Diffuse disease, *n*(%)	26 (60.5)
The presence of antinuclear antibodies, *n*(%)	43 (100)
Anti-Scl-70 antibodies, *n*(%)	24 (55.8)
Anti-PM/Scl antibodies, *n*(%)	7 (16.3)
Anti-centromeric antibodies, *n*(%)	10 (23.3)
Anti-NOR antibodies, *n*(%)	2 (4.7)
Anti-Ro52 antibodies, *n*(%)	11 (25.6)
RNA polymerase III antibodies, *n*(%)	1 (2.3)
Anti-Th/To antibodies, *n*(%)	1 (2.3)
Anti-Ku antibodies, *n*(%)	1 (2.3)
Organ involvement	
Digital ulcers, *n*(%)	14 (32.6)
Abnormal nailfold capillaries, *n*(%)	26 (60.5)
Telangiectasia, *n*(%)	12 (27.9)
Raynaud’s phenomenon, *n*(%)	41 (95.3)
Dysphagia, *n*(%)	10 (23.3)
Interstitial lung disease, *n*(%)	30 (69.8)
Pulmonary arterial hypertension, n(%)	10 (23.3)
Renal crisis, n(%)	1 (2.3)
*Systemic sclerosis-specific therapy*	
Current corticosteroids therapy, *n*(%)	16 (37.2)
Current corticosteroid dose, mg per day (recalculated to methylprednisolone)	0 (0–4)
Systemic steroids therapy, years	0 (0–3)
*Immunosuppressive treatment (currently or in the past)*	
Azathioprine, *n*(%)	5 (11.6)
Cyclophosphamide, *n*(%)	16 (37.2)
Methotrexate, *n*(%)	12 (27.9)
Mycophenolate mofetil, *n*(%)	5 (11.6)
*Internal medicine comorbidities*	
Hypertension, *n*(%)	18 (41.9)
Diabetes mellitus, *n*(%)	3 (7)
Hypercholesterolemia, *n*(%)	18 (41.9)
Internist medications	
Angiotensin-converting enzyme inhibitors or angiotensin receptor antagonists, *n*(%)	21 (48.8)
Statins, *n*(%)	13 (30.2)
Beta-blockers, *n*(%)	10 (23.3)
Diuretics, *n*(%)	10 (23.3)
Calcium channel blockers, *n*(%)	26 (60.5)

Categorical variables are presented as numbers (percentages), continuous variables as the median and interquartile range (25–75Q)

*n* number

Antinuclear antibodies were detected in all SSc patients. As expected, the most frequent were anti-topoisomerase I antibodies (Scl-70), found in 24 (55.8%) of them, followed by anti-centromere antibodies (ACA) and/or anti-Ro52 reported in nearly one-quarter of all patients. Other types of antibodies, such as anti-RNA polymerase III, anti-PM/Scl, anti-NOR, anti-Th/To, and anti-Ku, were less prevalent.

### Systemic sclerosis patients had higher serum TNFα levels

Table 2 compares the serum cytokine levels in SSc and control groups. TNFα was the only difference, 17% higher in SSc (*p* = 0.04). Diffuse and limited SSc phenotypes did not differ in studied cytokine concentrations; however, those on systemic steroids, thus representing a more severe disease form, had higher levels of IL-17 and FAP (Table 3).

**Table 2:** Serum cytokines levels of the subjects studied

	Systemic sclerosis patients *n* = 43	Controls *n* = 24	*p* value
Tumor necrosis factor α [pg/ml]	5.3 (3.5–8.2)	4.4 (3.5–4.4)	0.04*
Interleukin-6 [pg/ml]	3 (3–3)	3 (3–3)	0.61
Fibroblast activation protein [pg/ml]	30,520 (16,115.6–30,520)	30,520 (30,520–30520)	0.15
Interleukin-10 [pg/ml]	7.4 (2.6–7.4)	7.4 (0.8–7.4)	0.35
Interferon gamma [pg/ml]	5.6 (5.6–11.4)	5.6 (5.6–5.6)	0.09
Interlukin-17A [pg/ml]	1.1 (1.1–1.3)	1.1 (1.1–1.1)	0.44
Interlukin-4 [pg/ml]	14.6 (14.6–18.23)	14.6 (14.6–14.6)	0.6

Continuous variables are presented as the median and interquartile range (25–75Q)

*A statistically significant difference

**Table 3:** Comparison of cytokine levels between systemic sclerosis patients treated with corticosteroids and immunosuppressants vs. not receiving those medications at enrollment

Cytokine levels in patients treated with corticosteroids				
	Yes	No	*z*	*p*
Tumor necrosis factor α	6.56 (3.74;8.52)	4.39 (3.52;7.91)	0.403	0.6866
Intereukin-6	3 (3;6.66)	3 (3:3)	1.514	0.12993
Interleukin-17A	1.2 (1.06;2.02)	1.06 (1.06;1.06)	2.878	0.00531*
Interleukin-4	14.6 (11.55;16.42)	14.6 (14.6;18.2)	− 0,12	0.904
Interferon γ	5.56 (5.56;11.43)	5.56 (5.56;11.43)	0.387	0.69858
Interleukin-10	7.41 (4.89;7.41)	7.41 (2.59;7.41)	0.213	0.8313
Fibroblast activation protein	30,520 (11,764.63;30,520)	30,520 (30,520;30,520)	− 1,999	0.04564*

Cytokine levels in patients treated with immunosuppressants other than corticosteroids				
	Yes	No	*z*	*p*
Tumor necrosis factor α	5.33(4.39;11.59)	4.86(3.52;8.06)	0.792	0.428117
Intereukin-6	3(3;4.66)	3(3:3)	0.789	0.429849
Interleukin-17A	1.06(1.06;2.02)	1.06 (1.06;1.2)	0.129	0.897496
Interleukin-4	14.6(8.49;14.6)	14.6 (14.6;18.2)	− 0,983	0.325788
Interferon γ	5.56(5.56;11.43)	5.56 (5.56;8.5)	0.892	0.372204
Interleukin-10	7.41(0.51;7.41)	7.41 (7.41;7.41)	− 1,0513	0.293109
Fibroblast activation protein	30,520 (10,255.15;30,520)	30,520 (30,520;30,520)	− 1,0511	0.293233

Continuous variables are presented as the median and interquartile range (25–75Q)

*A statistically significant difference

### Measured serum cytokines were associated with basic laboratory test results but not systemic sclerosis clinical manifestations

As expected, there was a positive correlation between IL-6 and C-reactive protein levels (*r* = 0.321, *p* = 0.035). Additionally, we documented the association between TNFα and creatine concentrations in peripheral blood (*r* = 0.316, *p* = 0.038). Interestingly, elevated FAPs, defined as values above 16,115 pg/mL at baseline, were associated with a higher prevalence of anti-Ro52 antibodies.

On the contrary, studied cytokines were unrelated to clinical SSc manifestations, such as digital ulcers, abnormal nailfold capillaries, ILD, or PAH, and other comorbidities (data not shown). Interestingly, patients with higher IL-17, defined as values ≥ 1.06 pg/mL, were at 2.3 (95% CI 1.49–3.51, *p* = 0.009) OR of having decreased LVEF (< 67%).

### Higher circulating TNFα at baseline was associated with increased cancer risk, whereas higher IL-17 resulted in death in a 5 year follow-up

During the median five years of follow-up, four SSc patients were diagnosed with cancer; two had lung cancer, one had a squamous cell carcinoma of unknown origin, and one had breast cancer and multiple myeloma. Nine SSc patients died during the follow-up period. The causes of death included lung cancer (*n* = 2), renal crisis (*n* = 1), multiple-organ failure (*n* = 1), and in five cases, the reason for death was unknown. Interestingly, those with higher IL-17 levels, defined as values ≥ 1.388 pg/mL had 3.6 (95% CI 1.51–8.61, *p* = 0.004) OR of death. There was a significant difference in the presence of cancer depending on the level of TNFα (the Mann–Whitney *U* test, *p* < 0.01). Surprisingly, all malignant patients had elevated circulating TNF α ≥ 7.91 pg/mL at baseline. The probability of cancer in the SSc group with TNF α ≥ 7.91 pg/mL was 29%, whereas in those with TNFα < 7.91 pg/mL was 0% (*p* = 0.002).

Interestingly, IL-17 and FAP levels were higher in patients receiving oral steroids (Table 3). Other immunosuppressants (Table 3) or medications did not impact the cytokine levels (data not shown).

### Independent determinants of higher mortality in systemic sclerosis patients

A multiple linear regression model demonstrated that higher pulmonary artery systolic pressure (PASP), lower LVEF, and elevated CRP were the most potent independent determinants of higher mortality risk in the 5 year follow-up of SSc patients (Table 4).

**Table 4:** The multiple logistic regression model shows associations between laboratory and clinical variables and mortality risk in the 5 year follow-up

Parameter	β	SD	95% CI	OR	*p*
C-reactive protein [mg/L]	0.185	0.083	(1.022–1.416)	1.203	0.026
Left ventricle ejection fraction [%]	− 0.202	0.088	(0.688–0.97)	0.021	0.021
Pulmonary artery systolic pressure [mmHg]	0.252	0.125	(1.006–1.644)	0.045	0.045

*CI* confidence interval, *OR* odds ratio, *SD* standard deviation

### Elevated serum TNFα and IL-17 levels are associated with *TNFA* gene polymorphisms

Frequencies of particular *TNFA* genotypes in the SSc group were presented in our previous publication. They were not associated with patients' clinical characteristics. Contrarily, higher circulating IL-17 levels were observed in CT rs1799964 genotypes compared to TT carriers (*p* = 0.021). Moreover, the AG rs361525 genotype referred to the increased serum TNFα comparing GG carriers (*p* = 0.048). Furthermore, interestingly, both of them (CT rs1799964 and AG rs361525 genotypes) were associated with a higher frequency of cancer (two patients had lung cancer, one had planoepithelial carcinoma of unknown origin confirmed in histological analysis of lymph nodes).

## Discussion

Our study points to the critical role of TNFα in SSc pathology. First, it was the only cytokine higher in that disease; furthermore, all who developed cancer in 5-year follow-up had the highest levels of TNFα at baseline. Eventually, AG and GC rs361525 genotypes of *TNFA* gene polymorphism predicted higher serum TNFα and simultaneously a higher frequency of cancer.

TNFα is a pro-inflammatory cytokine released by various immune cells, including B and T lymphocytes, macrophages, neutrophils, fibroblasts, and NK cells. Its primary function is to protect against malignancies and infections [43]. It is also considered a fibrogenic cytokine, along with IL-1β, transforming growth factor beta, platelet-derived growth factor, and fibroblast growth factor. In SSc, elevated levels of those cytokines may contribute to the proliferation of fibroblasts and endothelial cells, leading to connective tissue and endothelium alterations, a primary hallmark of the disease [44]. Indeed, in our previous reports, we demonstrated that SSc patients present with endothelial dysfunction, prothrombotic state and increased oxidative stress [2, 45, 46].

Our results regarding TNFα are consistent with other research findings. Hagesawa et al. [30] conducted a study in 51 patients with limited cutaneous SSc and 30 patients with diffuse cutaneous SSc, showing increased serum TNFα levels associated with the diagnosis of pulmonary fibrosis. Kantor et al. reported similar results in 30 patients with definite SSc, noting higher spontaneous release of TNFα in patients with early diffuse cutaneous disease [31]. Our study found no relationship between TNFα and organ involvement, possibly due to the limited number of patients.

However, we found an association between the *TNFA* polymorphisms rs1799964 and rs361525 and cancer susceptibility in SSc patients without any other correlations to the laboratory and clinical characteristics. In turn, as we found in this report, AG and GC rs361525 genotypes may be related to higher serum TNF alpha, leading to increased cancer risk in this mechanism.

TNF α has a dualistic relation to malignancies. On the one hand, it acts as an antitumoral cytokine, but on the other, chronically elevated levels in low concentrations can promote tumor growth [47], which is consistent with our findings. Laboratory murine cancer models suggest that TNFα promotes malignancy by influencing both initiated cancer-developing cells and inflammatory cells in the microenvironment [48]. In clinical studies, elevated TNFα levels have been found in the blood of patients with renal, pancreatic, breast, and prostate cancers, interestingly in correlation with disease advancement and poor survival [43, 49]. Furthermore, it has been shown that high doses of recombinant TNFα are effective for treating melanoma [50] and soft tissue sarcomas [51] in isolated limb perfusion, where the agent penetrates local metastases but not the circulation. On the other hand, systemic administration of high TNFα doses has been associated with significant side effects, including hypotension and multiple organ failure [52]. On the contrary, an ongoing clinical trial exploring the removal of soluble TNFα receptors from the plasma of patients with advanced, refractory breast cancer using immunopheresis provides encouraging results [53].

TNFα inhibitors, such as infliximab, adalimumab, certolizumab, and etanercept are approved for treating chronic inflammatory diseases [54]. In experimental settings, those agents reduced collagen production and fibrosis and decreased vascular endothelial growth factor (VEGF) release, a proangiogenic factor associated with pulmonary arterial hypertension [54]. However, the clinical use of TNFα inhibitors in SSc needs to be evaluated in randomized clinical trials, as no ongoing trials have been reported to date. Encouragingly, there are case reports of successful infliximab administration in SSc patients who were refractory to conventional therapies [55, 56].

Another issue that merits comment in our data is the role of IL-17 in SSc prognosis. Surprisingly, patients with higher IL-17 in serum had an increased risk of death in a 5-year follow-up, representing a crucial finding of our study. Remarkably, levels of IL-17 and FAP were higher in patients on oral steroids, representing likely the more severe cases. Furthermore, higher IL-17 concentrations were associated with lower LVEF values, which also independently predicted, together with elevated CRP and PASP, increased mortality rate in multiple regression models.

Regarding IL-17, this pro-inflammatory cytokine is primarily produced by the Th17 cell subset in response to TGF-β and IL-23 and plays an essential role in host-defense mechanisms [25, 57, 58]. In SSc, IL-17 stimulates endothelial and epithelial cells and fibroblasts to secrete immunomodulatory factors [13, 25, 57]. IL-17 may play a role in autoimmune inflammatory diseases, such as systemic lupus erythematosus (SLE) [59]; however, data on its inhibition by mononuclear antibodies in that disease were ambiguous [60, 61].

Nevertheless, our results suggest that in SSc, IL-17 overproduction might worsen clinical prognosis, which has also been documented by Wei L et al. [25]. So far, no other studies with similar conclusions could support our findings. However, secukinumab, an approved IL-17 inhibitor for treating chronic inflammatory diseases, potentially ameliorated dermal fibrosis in mouse models of bleomycin-induced fibrosis [62]. It is noteworthy that IL-17 inhibitors in SSc are not, so far, approved by regulatory agencies, but a randomized clinical trial involving brodalumab has been undertaken (currently no longer recruiting) [13]. Nonetheless, a lack of published data exists regarding administering IL-17 inhibitors in SSc patients.

Our data indicate that the CT variant of rs1799964 in the *TNFA* gene may be associated with a predisposition to higher serum IL-17 levels, suggesting possible cross-talk between TNFα and IL-17 regulation. That is also a novel and exciting finding of our study. Additionally, an intriguing observation regards the potential influence of steroid administration on elevated IL-17. That raises an important question on the impact of chronic steroid therapy on the SSc prognosis. However, we are not able to exclude that steroid-treated patients were more severely ill and mutually had higher IL-17 levels.

### Study limitation

The main limitation of our study is the low number of SSc patients, which may impact genetics the most. However, we used Hardy–Weinberg equilibrium with linkage disequilibrium to overcome that limitation. Furthermore, SSc is a rare disease, and each observation is valuable.

## Conclusions

In summary, SSc is characterized by elevated serum TNFα, independently of organ involvement. Furthermore, elevated TNFα might be linked to cancer and is related to the AG genotype of rs361525 *TNFA* polymorphism in that disease. On the other hand, higher IL-17 might be associated with increased death risk. Future research, however, is needed to validate our findings.

## Data Availability

The data presented in this study are available on request from the corresponding author. The data are not publicly available due to patients’ origin.
